# Supplementary material for: Predicting visual function by interpreting a neuronal wiring diagram
Source: Nature. 2024 Oct 2;634(8032):113–23. doi: 10.1038/s41586-024-07953-5 (PMC11446822; doi:10.1038/s41586-024-07953-5)
Supplement: Supplementary file 6 — CRF and ERF predictions for individual TmY4 and TmY9 cells. Analogous to Supplementary Data 3, but for TmY target types. Shown are the top four monosynaptic pathways, the strongest pathway passing through each of the top ten intermediary types (ranking from Extended Data Fig. 7), and the trisynaptic pathway Tm1–TmY–Dm3–TmY (see the section entitled Prediction of spatial normalization). [file 41586_2024_7953_MOESM6_ESM.zip › DataS4/TmY9q/720575940639210037.pdf]

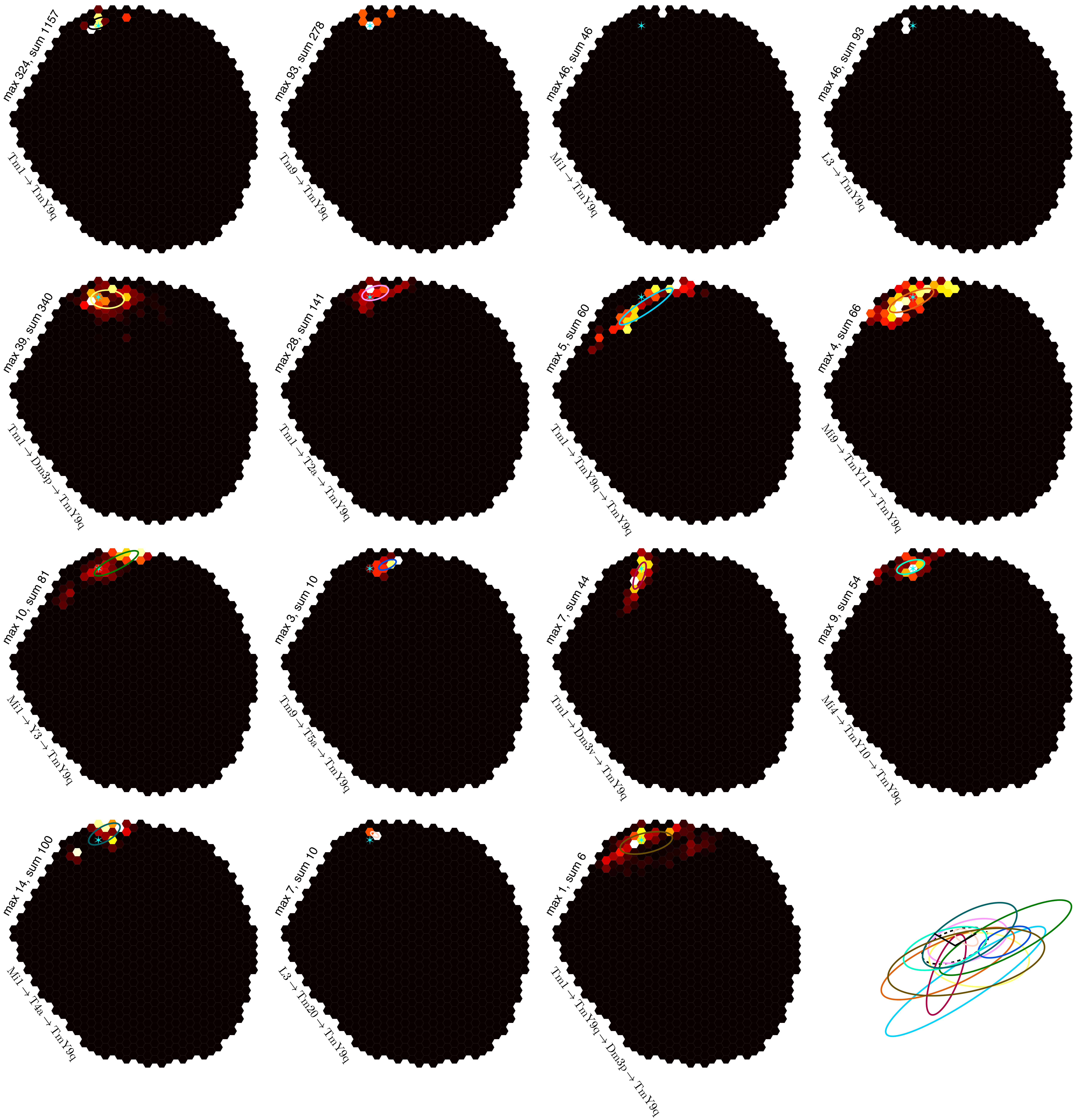
max 324, sum 1157
Tm1 → TmY9q
max 93, sum 278
Tm9 → TmY9q
max 46, sum 46
Mi1 → TmY9q
max 46, sum 93
L3 → TmY9q
max 39, sum 340
Tm1 → Dm3p → TmY9q
max 28, sum 141
Tm1 → T2a → TmY9q
max 5, sum 60
Tm1 → TmY9q → TmY9q
max 4, sum 66
Mi9 → TmY11 → TmY9q
max 10, sum 81
Mi1 → Y3 → TmY9q
max 3, sum 10
Tm9 → T5a → TmY9q
max 7, sum 44
Tm1 → Dm3v → TmY9q
max 9, sum 54
Mi4 → TmY10 → TmY9q
max 14, sum 100
Mi1 → T4a → TmY9q
max 7, sum 10
L3 → Tm20 → TmY9q
max 1, sum 6
Tm1 → TmY9q → Dm3p → TmY9q
